# Early Results of Mechanochemical Ablation with Flebogrif® in great Saphenous Vein Insufficiency: does Polidocanol Concentration Affect Outcome?

**Published:** 2020-02-20

**Authors:** RP Ammollo, A Petrone, AM Giribono, L Ferrante, L del Guercio, UM Bracale

**Affiliations:** 1 Vascular Surgery Unit, Department of Public Health, University Federico II of Naples, Naples, Italy; 2 Department of Medicine and Health Science, University of Molise, Campobasso, Italy

## Abstract

**Background:**

Flebogrif® (Balton, Poland) is a novel mechanochemical ablation (MOCA) device for saphenous vein insufficiency. It combines endothelial damage performed by radial retractable cutting hooks together with chemical ablation through sclerosant injection of 3% polidocanol foam according to its IFU. The objective of this study is to evaluate Flebogrif’s efficacy in terms of recanalization rate and recurrence by varying polidocanol foam concentrations.

**Methods:**

We performed 24 MOCAs on 23 patients with Flebogrif® between January and May 2019. In 12 cases the polidocanol foam was prepared at a 3% concentration, and in another 12 at 1.5%. Great saphenous vein (GSV) recanalization and truncular recurrence were evaluated at 1 and 3 months with a Duplex Ultrasound Anatomy (DUS) examination.

**Results:**

At 1- and 3-month follow-ups, none of the 14 patients treated with the polidocanol 3% foam were observed to have had great saphenous vein GSV recanalization and truncular recurrence. Only 2 of the 14 (14.3%) cases treated with polidocanol 1.5% foam showed evidence of recanalization within the first centimetres from the sapheno-femoral junction (p > .05). All patients experienced clinical benefits without recurrence of symptoms.

**Conclusion:**

MOCA with Flebogrif® is a safe, relatively inexpensive and effective alternative to standard methods in the treatment of saphenous insufficiency with encouraging short-term results. Despite our relatively small patient sample, no statistical significance in evidence of recurrence in the group of patients treated with 3% foam and those treated with 1.5% foam was noted. Longer term analysis of GSV patency and recurrence is necessary to further evaluate Flebogrif’s impact and actual indications in the treatment of chronic venous disease.

## I. BACKGROUND

Endovascular techniques for the treatment of saphenous vein insufficiency have been increasing in number and complexity over the last years. Among them, endovenous laser ablation (EVLA) and radiofrequency ablation (RFA) are now considered first-choice treatments, according to the latest guidelines for truncal ablation.^1^

In the already crowded phlebological panorama, mechanochemical ablation (MOCA) is a recently introduced mechanism which combines chemical damage through sclerosant foam injection with an endothelial spasm performed by a rotating wire or radial cutting hooks commercially patented as Clarivein® (Merit Medical, Utah, USA) and Flebogrif® (Balton, Poland), respectively. The endothelial and medial mechanical damage is purported to enhance the penetration of the sclerosant in the vessel wall and the subsequent vasoconstriction as proven in ex vivo and animal models.^2^,^3^ Thus it is reasonable to assume that recanalization rates would be lower than with mechanical or chemical ablation treatments alone.

### Clarivein

Being the first MOCA device ever produced and studied, Clarivein® has experienced broad success and wide application, including in the treatment of venous leg ulcers.^4^,^5^ It has been also compared to other techniques such as EVLA and RFA with satisfying results, proving a high safety profile and low recanalization rates.^6^,^7^ Results from a randomized controlled trial published in December 2019 confirm shorter operative time, lower rates of postoperative phlebitis and significantly shorter time to return to work following treatment^8^.

### Flebogrif

As of yet, evidence is still scarce on Flebogrif. Zubilewicz et al.^9^ reported a 93% occlusion rate at three months and only 1 deep vein thrombosis complication in a cohort of 200 patients. Unlike Clarivein®, no study has yet been conducted which observes the histological effects of Flebogrif cutting hooks on the endothelium and whether it indeeds increases sclerosant penetration in the vessel wall, nor has a comparison been made on its benefit over other comparable techniques.

According to the Instructions for Use, both Clarivein and Flebogrif® require a preparation of 2–3% polidocanol foam to be injected while the system is retracted though the trunk to be ablated. On the other hand, the use of 1 or 1.5% foam concentration even in large vessels such as the great saphenous vein at the sapheno-femoral junction (SFJ) is considered safe as reported in the literature.^10^

The objective of this study is to evaluate Flebogrif’s efficacy in terms of the recanalization rate and recurrence based upon varying the polidocanol foam concentrations.

## II. METHODS

Among patients with primary varicose disease we included those reporting chronic venous disease symptoms (leg heaviness and swelling, pruritus, etc.), evidence of reflux at the SFJ; a linear GSV - without big, tortuous truncular collaterals - and a diameter of the GSV at level of the SFJ diameter no bigger than 60 mm.

**Figure 1:**
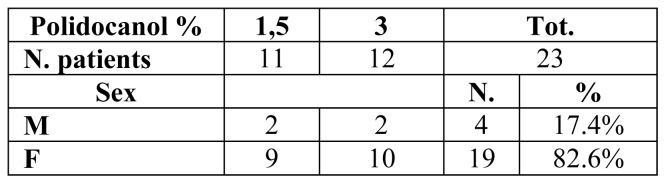
Distribution of patients between the two groups: 1.5% and 3% polidocanol foam concentrations.

Based upon these criteria, we selected 23 suitable patients and performed 24 MOCAs with Flebogrif® between January and May 2019. In 12 cases polidocanol foam was prepared at a 3% concentration, and in another 12 at a 1.5% concentration (figure 1).

**Figure 2:**
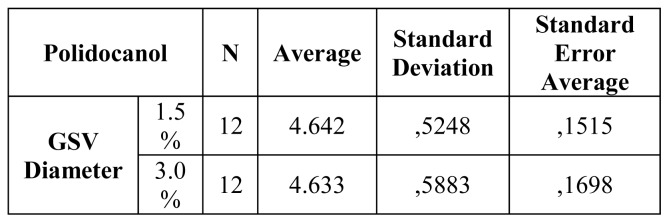
Comparison between the two groups according to GSV diameter at 2 cm from SFJ.

No statistical significance regarding the diameter of GSV at 2 centimeters from SVJ (Figure 2) existed. Thus, GSV recanalization and truncular recurrence were analysed at 1 and 3 months with a DUS examination. Statistical analysis was performed with IBM SPSS.

## III. RESULTS

**Figure 3:**
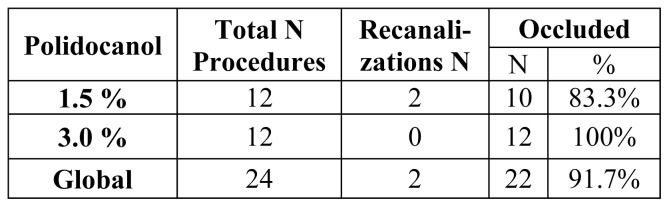
Occlusion rates at 3 months.

At 1-and 3-month follow-ups, no GSV recanalization and truncular recurrence in any of the 14 patients treated with polidocanol 3% foam was observed. Only in 2 of the 14 (14.3%) cases treated with polidocanol 1.5% foam was there evidence of recanalization in the first centimeters from the SFJ (figure 3).

**Figure 4:**
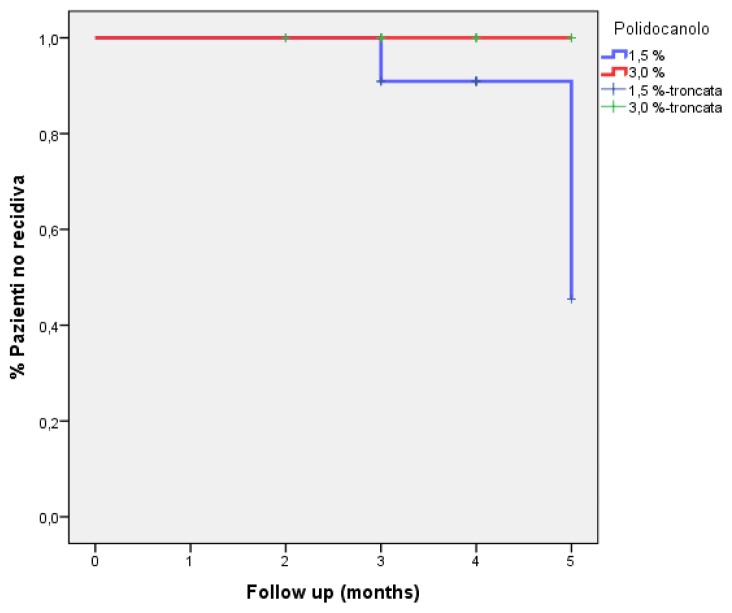
Kaplan-Meyer analysis of follow-up.

Follow-up data were also processed through Kaplan-Meyer analysis showing no statistical significance between the two groups in the first 3 months of follow-up (figure 4). All patients experienced good aesthetical results with no recurrence of symptoms.

## IV. DISCUSSION

Over the last few decades, the undiscussed role of classical surgical saphenous ablation in the different forms of *stripping* has been widely called into question, although the short groin incision – with limited risk of lymphocele and excellent cosmetic results – and mandatory isolation of the saphenous nerve at the medial malleolus, have sensitively limited its major complication rates.^11^

The progressive trend of abandonment of such surgical procedures started in the United States since the introduction of endovascular thermal techniques, initially without solid proof of benefits and a non-negligible rate of complication including thermal injury of the skin, deep vein thrombosis and recanalization.^6^

Already in the 2011 *Clinical practice guidelines of the Society for Vascular Surgery and the American Venous Forum*, surgery is restricted to patients with large, tortuous and superficial saphenous veins or to those having aneurysmal enlargement at SFJ.^12^ As well, the 2017 *American College of Phlebology* Guidelines also recommend endovenous thermal ablation as the preferred treatment for saphenous and accessory saphenous vein incompetence, and advise the use of mechanical/chemical ablation with Clarivein to treat truncal venous reflux, but with a lower level of evidence.^13^ In parallel, ultrasound-guided foam sclerotherapy (UGFS) has reached a prominent position among the saphenous ablation techniques being considered as safe and effective a procedure as thermal techniques.^12^,^13^

According to the latest Guidelines from the *European Society for Vascular Surgery* (June 2015), RFA and EVLA are preferred over both surgery and foam sclerotherapy with an evidence of Grade I, Level A.^14^ Despite all these recommendations, there is still a lack of evidence of exhaustive long-term results comparing open surgery, foam sclerotherapy and endovascular procedures, giving univocal solutions or shared work-up algorithms for the treatment of saphenous insufficiency. According to the LARA and RECOVERY studies and recent reviews,^15^–^17^ RFA is equivalent to EVLA in terms of occlusion rates, however it causes less post-operative pain and bruising with a faster return to normal activities,

In several studies^18^–^20^ UGFS appear less effective than EVLA and/or RFA. A recent RCT of an eight-year follow-up claims that surgical stripping has a technically better outcome in terms of recurrence of GSV and SFJ reflux than UGFS in the long term, and that long-term follow-up suggests significant clinical progression of venous disease as measured by VCSS in both groups, but less so after surgery.^21^

Most of the studies available regarding the follow-up of mechanochemical ablation (all of which are on Clarivein®) are in the short-term and report good results, short operative time, high patient acceptance, low peri-procedural pain and no saphenous nerve injury.^4^,^22^,^23^ This is also confirmed when MOCA is compared to EVLA and RFA.^6^,^7^

Despite the absence of evidence on Flebogrif**®** in the short and mid-terms and no comparisons to any other truncal ablation techniques yet existing, in our experience Flebogrif**®** appears to have the same advantages as those observed for its counterpart Clarivein**®**, allowing for short, painless procedures with no need of tumescent anaesthesia. The operation can also be performed in an out-patient setting, has a fast recovery period and overall lower costs, especially if compared to EVLA or RFA.

The limitations of this study are the small number of cases and the short follow-up period. Also, the Flebogrif® procedures were performed in patients with relatively small incompetent GSV, and no comparison with other techniques has been made.

Further investigation is required to broaden the number of treated cases, extend the follow-up period and should include a comparison with surgical or thermal ablation techniques.

## V. CONCLUSION

MOCA with Flebogrif**®** is a safe, relatively inexpensive and effective alternative to standard methods (i.e. surgical treatment, thermal ablation, etc.) in the treatment of saphenous insufficiency, with encouraging results reported in the short term. Despite our relatively small patient sample, no statistical significance was found in evidence of recurrence in the group of patients treated with 3% foam and in those treated with 1.5% foam. Long term analysis of GSV patency and recurrence is necessary to evaluate its impact and actual indications in the treatment of chronic venous disease.
